# Impact of the neutrophil response to granulocyte colony-stimulating factor on the risk of hemorrhage when used in combination with tissue plasminogen activator during the acute phase of experimental stroke

**DOI:** 10.1186/1742-2094-11-96

**Published:** 2014-05-27

**Authors:** Sophie Gautier, Thavarak Ouk, Madjid Tagzirt, Catherine Lefebvre, Maud Laprais, Olivier Pétrault, Annabelle Dupont, Didier Leys, Régis Bordet

**Affiliations:** 1 EA 1046 - Département de Pharmacologie médicale, Université de Lille 2 - Faculté de Médecine, 1 place de Verdun, Lille cedex F-59037, France; 2 EA2693 - Laboratoire d’Interface sang-vaisseaux et de réparation cardiovasculaire, Centre Hospitalier Universitaire, 1 place de Verdun, Lille cedex F-59037, France; 3 EA 2691 - Service de Neurologie et Pathologies Vasculaires, Institut de Médecine Prédictive et de Recherche Thérapeutique, 1 place de Verdun, Lille cedex F-59037, France

**Keywords:** Stroke, tPA, G-CSF, Neutrophil, Hemorrhage, Vascular endothelium

## Abstract

**Background:**

Granulocyte colony-stimulating factor (G-CSF) is a pharmacologic agent inducing neutrophil mobilization and a new candidate for neuroprotection and neuroregeneration in stroke. Its effects when used in combination with tissue plasminogen activator (tPA) were explored during the acute phase of ischemic stroke.

**Methods:**

We used a middle cerebral artery occlusion (MCAO) model of cerebral ischemia, associated with treatment with tPA, in male spontaneously hypertensive rats (SHR). Granulocyte colony-stimulating factor (G-CSF; 60 μg/kg) was injected just before tPA. Neutrophil response in peripheral blood and in the infarct area was quantified in parallel to the infarct volume. Protease matrix metallopeptidase 9 (MMP-9) release from circulating neutrophils was analyzed by immunochemistry and zymography. Vascular reactivity and hemorrhagic volume in the infarct area was also assessed.

**Results:**

Twenty four hours after ischemia and tPA, G-CSF administration induced a significant increase of neutrophils in peripheral blood (*P* <0.05). At 72 hours post-ischemia, G-CSF was significantly associated with an increased risk of hemorrhage in the infarct area (2.5 times more likely; *P* <0.05) and significant cerebral endothelium-dependent dysfunction. *Ex vivo,* an increased MMP-9 release from neutrophils after tPA administration correlated to the increased hemorrhagic risk (*P* <0.05). In parallel, G-CSF administration was associated with a decreased neutrophil infiltration in the infarct area (-50%; *P* <0.05), with a concomitant significant neuroprotective effect (infarct volume: -40%; *P* <0.05).

**Conclusions:**

We demonstrate that G-CSF potentiates the risk of hemorrhage in experimental stroke when used in combination with tPA by inducing neutrophilia. This effect is concomitant to an increased MMP-9 release from peripheral neutrophils induced by the tPA treatment. These results highlight the potential hemorrhagic risk of associating G-CSF to thrombolysis during the acute phase of stroke.

## Introduction

Granulocyte colony stimulating factor (G-CSF) is a hematopoietic cytokine initially used in the treatment of neutropenia for its effects on proliferation and maturation of the granulocyte cell line.

Recently, G-CSF displayed a neuroprotective effect when used during experimental stroke by both decreasing the infarct size and improving motor function recovery [1-3]. Several clinical studies using G-CSF during the acute and subacute phase of ischemic stroke have now been reported [4]. At the acute phase of ischemic stroke, the question of combining G-CSF with tissue plasminogen activator (tPA), used for thrombolysis, is raised. tPA is currently the only licensed drug for acute stroke, allowing reperfusion of the infarct area. However, the tPA-therapeutic window is limited due to induced-hemorrhages [5]. Given the role played by polymorphonuclear neutrophils (PMN) in the physiopathology of these post-tPA hemorrhagic complications [6], the objective of this work was to analyze the impact of G-CSF on the risk of hemorrhage when used in combination with tPA during the acute phase of an experimental ischemic stroke.

## Methods

All animal experiments were performed in strict accordance with the guidelines published by the International European Ethical Standards (86/609-EEC) and the French Department of Agriculture (décret 87/848). Spontaneously hypertensive rats (SHRs; 10-week-old male, weighing between 270 and 320 g, from Elevage Janvier Labs, Le Genest Saint Isle, France) were used in this study. Experimental data were monitored by using a blinded investigator for group allocation.

### Surgical procedure and design of the study

Cerebral infarction was induced by intraluminal middle cerebral artery occlusion (MCAO) as previously described [7]. Briefly, rats were anesthetized by an intraperitoneal injection of chloral hydrate (300 mg/kg, 1.7 ml). Body temperature was maintained at 37 ± 0.5°C throughout the surgery. The right carotid arteries were exposed through a midline cervical incision and the common carotid and external carotid arteries were ligated with a silk suture. An aneurysm clip was placed across the internal carotid artery and an arteriotomy was made in the common carotid artery stump, allowing the introduction of a monofilament nylon suture with its tip rounded by flame heating. The suture was gently advanced into the internal carotid artery and passed into the intracranial circulatory system as far as in the narrow lumen at the start of the middle cerebral artery (MCA). After one hour the suture was carefully removed to allow reperfusion. To reproduce the conditions of thrombolysis and induced hemorrhages, tPA 10 mg/kg (6 mL/kg) was administered after its *in vitro* application on a clot (made from 0.2 mL of autologous blood sampled by jugular vein during the surgery and left in the open air for five hours, allowing thrombus formation) for 30 minutes. The resulting solution (mainly contained plasmin) was collected and infused five hours after restoring cerebral blood flow [7]. All rats underwent MCAO and tPA treatment.

Two groups were randomly formed before surgery (n = 20 per group divided into evaluation of infarct, hemorrhage, and neutrophil infiltration (n = 13) and vasoreactivity analysis (n = 7)) and received G-CSF (60 μg/kg; 30 μL, Amgen, Neuilly, France) or vehicle (0.9% saline, 30 μL), subcutaneously administered just before administration of the tPA solution, five hours after the reperfusion. Sham-operated animals were treated identically, except that the MCA was not occluded (n = 4 for vehicle and n = 4 for G-CSF). Blood samples were collected before surgery, 24 and 72 hours later, to count leucocytes and PMNs (Machine XE 2100, Sysmex, Mississauga, Canada).

### Evaluation of infarct and hemorrhage

Seventy-two hours after restoring blood flow, rats were perfused with 60 mL of fresh saline just before sacrifice. The brains were rapidly removed and frozen. Coronal, 20 μm-thick slices were taken from 12 levels, according to Paxinos and Watson's stereotaxic atlas. Infarct volume (in mm^3^ and corrected for edema) was quantified by digital integration of the respective ischaemic areas on all sections in a given animal. Hemorrhages were assessed by blind histological evaluation on three defined sections (+0.48, -0.92, and -3.30 mm relative to the bregma). The incidence of hemorrhagic transformation (HT) was scaled according to a previously described method [8]: 0 = no hemorrhage; 1 = multiple, macroscopically visible hemorrhages seen as petechiae; and 2 = hematoma. The severity of the HT was deemed to correspond to the number of petechial hemorrhages or hematoma per infarct area. *In vivo* magnetic resonance imaging was performed to document hemorrhages and infarct in a set of animals (n = 6 per group) just before sacrifice in a 7-Tesla, narrow-bore small animal imaging system (Biospec 70/20 USR, Bruker Biospin, Wissembourg, France). We acquired two-dimensional, T2-weighted images using a rapid acquisition with refocused echoes pulse sequence (turboRARE; relaxation time: 2500 ms; echo time: 65 ms; field of view: 4 × 4 cm; matrix: 256 × 256, RARE factor: 8).

### Myeloperoxidase immunohistochemistry

Neutrophil infiltration was quantified after 72 hours of blood flow restoration by assaying myeloperoxidase (MPO), an enzyme expressed by neutrophil cells, using a rabbit polyclonal anti-MPO primary antibody (DAKO, Les Ulis, Franceand revealed by treatment with an avidin: biotinylated enzyme complex (PK-6100, ABC kit, Vector Labs, Burlingame, United States), as previously described [9]. Neutrophil infiltration was assayed in a coronal slice (+0.48 mm relative to the bregma) that featured positive cells on six adjacent 1 mm^2^ fields in the ischaemic zone (representative of 70 to 90% of the ischaemic tissue in the slice, located in cortical, subcortical, and striatal structures). We used brain sections from sham rats as a control.

### Vasoreactivity analysis

Endothelium-dependent relaxation was assessed after 72 hours of blood flow restoration in a Halpern arteriograph (Living Systems Instrumentation, Burlington, Vermont, United States) [10]. We used a proximal segment of the right MCA perfused with oxygenated Krebs solution and maintained at 37°C and pH 7.4. The experiment itself was performed under no-flow conditions. The lumen diameter was measured using image analysis. The relaxant dose-response curve for acetylcholine (Ach) was determined by stepwise, cumulative addition (from 0.001 to 10 μM Ach). Control groups (n = 4 for vehicle and n = 4 for G-CSF) were normotensive Wistar-Kyoto male rats, which served as a control group under physiological conditions [11]. Neutrophil depletion was induced by the intravenous administration of vinblastine (0.5 mg/kg EG labo, Boulogne-Billancourt, France) four days before the vasoreactivity analysis (n = 4). Relaxant responses were expressed as the percent increase in the pre-constricted artery diameter.

### Matrix metallopeptidase 9 (MMP-9) release from neutrophil degranulation

Peripheral neutrophil degranulation and MMP-9 release was investigated after *ex vivo* tPA administration, according to a previously described method [12]. Shortly after, neutrophils were isolated from the blood of rats submitted to MCAO alone or MCAO and G-CSF administration after 24 or 72 hours reperfusion (n = 10 in each group) versus control groups (n = 10) using a standard protocol [13]. After isolation, purified neutrophils were resuspended at a density of 1.10^6^ cells/mL and 400 mL of the cell suspension was seeded per well into 24-well plates. Neutrophils were allowed to rest for 90 minutes at 37°C, 5% CO_2_. The cells were then washed twice with phosphate buffer saline (PBS, Gibco BRL, Invitrogen, Cergy-Pontoise, France) and cultured in Roswell Park Memorial Institute (RPMI)-1640 medium with L-glutamine and sodium pyruvate (Gibco BRL) for another 30 minutes with PBS (control condition) or tPA (6.5 μmol/L). After stimulation, neutrophil-conditioned medium was collected, centrifuged (12 minutes at 14000 rpm), and the supernatant was stored at -80°C until analysis. The MMP-9 enzyme secreted by rat neutrophils was analyzed on gelatin zymography. Samples were mixed with an equal volume of 2 × sample buffer (which consisted of 10% sodium dodecyl sulfate (SDS), 10% glycerol, 0.5 M Tris-Hydrochloride (Tris-HCL), and 0.1% bromophenol blue at pH 6.8) and then added to 10% SDS-polyacrylamide gels (SDS-PAGE) co-polymerized with gelatin (1%) as the substrate for two hours. Following electrophoresis, gels were renatured in 2.5% Triton X-100 (Sigma-Aldrich, Saint Quentin, France) for 45 minutes at room temperature. The gels were then incubated at 37°C overnight in developing buffer (which consisted of 50 mM Tris-HCl, 0.2 M NaCl, and 5 mM CaCl_2_). Gels were stained with Coomassie Brilliant Blue R-250 (Bio-Rad, Marne la Coquette, France). Gelatinase activities were visualized as white bands on the blue background of the gels.

Direct MMP-9 release in the brains was assessed by immunochemistry [3]. MMP-9 positive signals were observed on 20-μm thick frozen coronal sections of brains from rats submitted to MCAO and tPA treatment and receiving vehicle or G-CSF alone at 24 and 72 hours versus control (n = 5 in each group). Diluted rabbit anti-rat MMP-9 antibody was used at a ratio of 1:400 (ab7299; Abcam; Cambridge, Massachusetts, United States) and incubated overnight at 4°C after the tissues were blocked for one hour in blocking solution containing PBS, 0.3% Triton X-100, 1% bovine serum albumin (BSA, Sigma-Aldrich, Saint Quentin, France), and 5% normal donkey serum (Clinisciences, Nanterre, France). The primary antibody was diluted in PBS containing 0.3% Triton X-100, 1% BSA, and 2% normal donkey serum. Sections were subsequently incubated with donkey anti-rabbit secondary antibody diluted at a ratio of 1:500 (Alexa Fluor 488 Dye, molecular probes, Invitrogen, Cergy-Pontoise, France) in PBS containing 0.3% Triton X-100, and 1% BSA for one hour, then washed with PBS and mounted with Vectashield mounting media for fluorescence (Vector labs, Burlingame, United States). Immunostaining was visualized with a fluorescent microscope (confocal Laser Scanning Microscopy 7 live, Zeiss, Fougères, France). Negative control sections were used without the primary antibody.

### Statistical analysis

All values were expressed as the mean ± standard error of the mean (SEM). We performed one-way analysis of variance (ANOVA) or Student’s *t*-test followed by *post hoc* protected Fisher’s least significant difference test when data were normally distributed (Kolmogorov-Smirnov test, *P* >0.05) and non-parametric tests, with Kruskal-Wallis and Mann-Whitney tests for comparisons of three (or more) non-related and two non-related groups, respectively. For comparisons of two related samples (such as repeated measurements on a single sample) we used the Wilcoxon signed-rank test. Results expressed as frequencies were compared using a Pearson’s chi-squared test. All statistical analyses were performed with SAS software (version 9.3, SAS Institute Inc., Cary, North Carolina, United States). *P* <0.05 was considered to indicate statistical significance.

## Results

### Physiological parameters

The mortality rate before 72 hours of reperfusion was the same between groups (n = 3 in vehicle group compared to n = 4 in G-CSF group). There were no differences in physiologic parameters (weight, blood pressure, temperature, pH, PaCO2, and PaO2) between the control and G-CSF administered groups before, during, and after the surgical procedure (data not shown). The blood parameters (prothrombin time and platelets) were not significantly different between the groups at baseline and at the end of the protocol.

### Neutrophil response to G-CSF treatment

The neutrophil count in the peripheral blood showed a significant increase in neutrophils after MCAO and tPA at 24 hours of reperfusion (*P* <0.05) and was significantly higher when G-CSF was administered (*P* <0.0001). This increase is still present but no more significant at 72 hours post-ischemia (Table 1).

**Table 1:** **Neutrophil counts on peripheral blood before surgery and after 24 h and 72 h of reperfusion in rats submitted to ischemia/reperfusion and tPA and treated by vehicle (saline 0.9**%**) or granulocyte colony stimulating factor (G-CSF 60 μg/kg; 30 μL)**

**Neutrophils (/mm**^**3**^**)**	**Vehicle (n = 9)**	**G-CSF (n = 10)**
Before ischemia	1808 ± 665	1645 ± 229
24 hours after ischemia	2993 ± 350*	6626 ± 1570*#
72 hours after ischemia	865 ± 444	1176 ± 587

Mean ± SEM. *p < 0.05 vs basal state; #p < 0.0001 vs vehicle. **G-CSF,** Granulocyte colony stimulating factor; tPA, tissue plasminogen activator, SEM standard error of the mean.

### Effect of G-CSF treatment on the risk of hemorrhage

**Figure 1:**
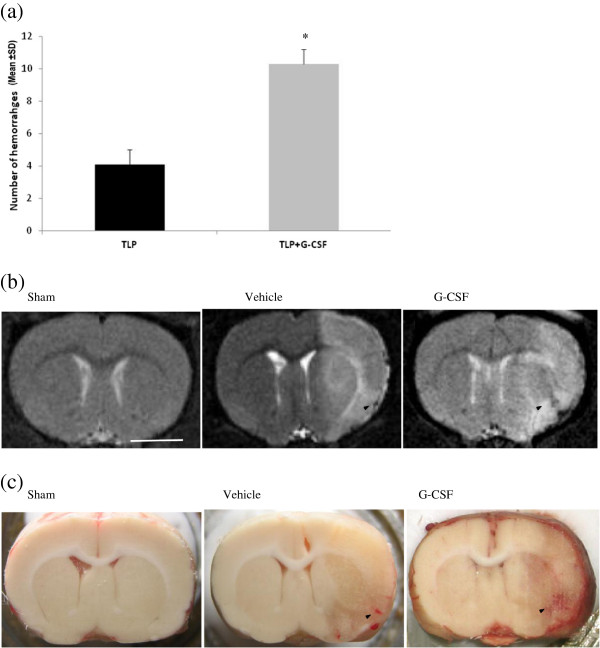
**Effect of subcutaneous administration of G-CSF (60 μg/kg; 30 μL) or vehicle (NaCl 0.9%, 30 μL) on the risk of hemorrhages. (a)** Number of hemorrhages in the infarct area (mean ± SD). **(b)** Hemorrhages were seen *in vivo* on T2-weighted MRI images. **(c)** Macroscopically visible on histological sections as petechiae or hematoma in the infarct area . Rats were all submitted to ischemia/reperfusion, tPA treatment and 72 hours of reperfusion. Scale bar = 500 μm. *P <0.05. G-CSF, granulocyte colony stimulating factor; MRI, magnetic resonance imaging; tPA, tissue plasminogen activator, SD standard deviation.

G-CSF was associated with a worsened risk of hemorrhage (Figure 1). Hemorrhages were more frequent (*P* <0.05) and more severe (*P* <0.05) in the G-CSF group compared to the vehicle group (Table 2).

**Table 2:** **Histologic examination of incidence and severity of intracerebral hemorrhages after 72 h of reperfusion in rats submitted to ischemia/reperfusion and tPA and treated by vehicle (saline 0.9**%**) or granulocyte colony stimulating factor (G-CSF 60 μg/kg; 30 μL)**

	**Histologic score**			**Severity**
	**0 = no hemorrhage (n)**	**1 = macroscopically visible hemorrhages (n)**	**2 = hematoma (n)**	**Mean number of petechial hemorrhages ± SEM**
Vehicle (n = 9)	1	8	0	4.1 ± 0.9
G-CSF (n = 10)	0	10	4*	10.3 ± 1.8 *

*p < 0.05 vs vehicle. **G-CSF,** Granulocyte colony stimulating factor; tPA, tissue plasminogen activator, SEM standard error of the mean.

### Effect of G-CSF treatment on endothelial function of cerebral vessels

**Figure 2:**
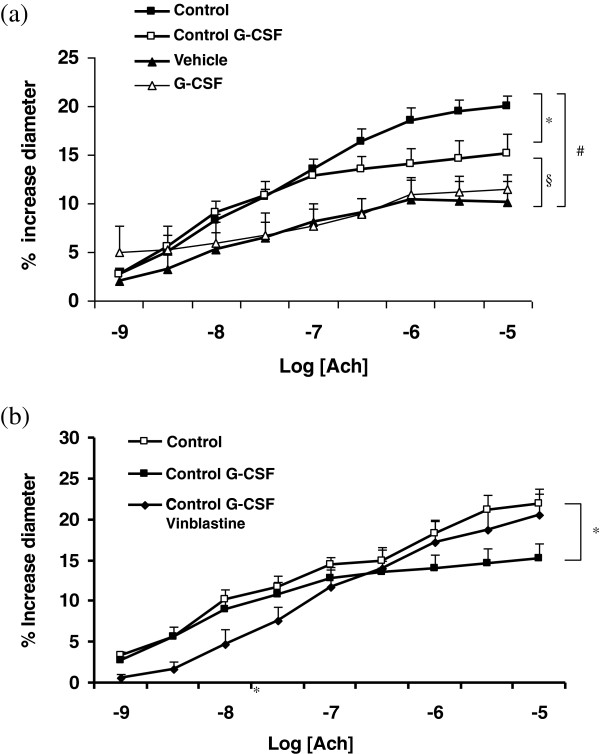
**Effect of subcutaneous administration of G-CSF (60 μg/kg; 30 μL) or vehicle on the endothelium-dependant function of the middle cerebral artery. (a)** Dose-response to acetylcholine after ischemia/reperfusion and thrombolysis. Endothelial relaxation was expressed as percentage of change in diameter of pre-constricted arteries measured after 72 hours of reperfusion. **(b)** Dose response to acetylcholine after vinblastine pretreatment in control rats. Vinblastine was used for neutrophil depletory and administered four days before the administration of G-CSF. Values are mean ± SEM. *P <0.05 versus control; § *P* <0.05 versus control G-CSF; #*P* <0.001 versus control. Ach, acetylcholine; G-CSF, granulocyte colony stimulating factor.

In control animals, G-CSF induced a significant alteration of the MCA endothelial function in response to an increasing dose of Ach (Figure 2a; *P* <0.05). Treatment with vinblastine (a non-specific neutrophil-depleting agent) four days before G-CSF administration prevented the observed endothelial dysfunction (Figure 2b; *P* <0.05). In animals submitted to MCAO and tPA the MCA endothelial function was significantly altered by the conditions (*P* <0.001) without the additive effect of treatment with G-CSF.

### Effect of G-CSF treatment on neutrophil infiltration and cerebral infarct size

**Figure 3:**
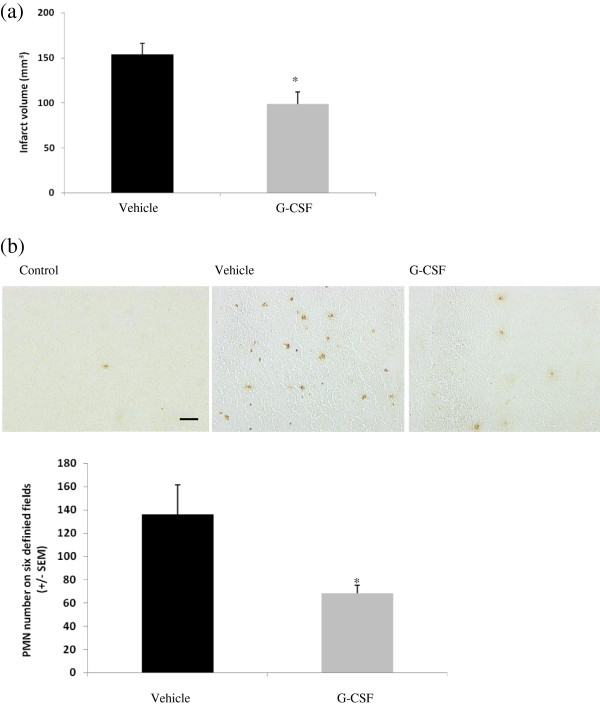
**Effect of subcutaneous administration of G-CSF (60 μg/kg; 30 μL) or vehicle (NaCl 0.9%, 30 μL). (a)** on infarct volume (corrected for edema). Volumes are expressed in mm3 (mean ± SEM). *P <0.05; **(b)** on neutrophil infiltration in infarct area. Infiltration was quantified by counting positive cells to anti-myeloperoxidase antibody on six adjacent fields of 1 mm^2^ on ischemic zone. All rats underwent ischemia/reperfusion, tPA treatment, and 72 hours of reperfusion. Values are mean ± SEM. *P <0.05. Scale bar: 100 μm. G-CSF, granulocyte colony stimulating factor; SEM standard error of the mean; PMN, polymorphonuclear neutrophils.

G-CSF, used in combination with tPA, in our MCAO model was associated with a significant decrease in neutrophil infiltration into the infarct area (68.5 ± 6.9 positive cells compared to 136.2 ± 25.2 in the vehicle group, *P* <0.05; Figure 3b). This effect was parallel to a neuroprotective effect as it induced a significant reduction in infarct size (98.71 ± 13.27 mm^3^ compared to 153.72 ± 12.25 mm^3^ in the vehicle group, *P* <0.05; Figure 3a).

### Effect of G-CSF treatment on MMP-9 release from neutrophil degranulation

**Figure 4:**
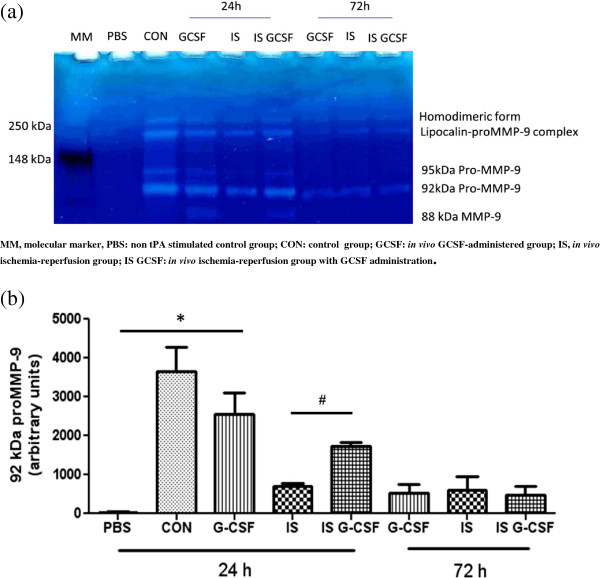
**MMP-9 release from peripheral neutrophil degranulation of purified rat neutrophils after 30 minutes *ex vivo *tPA stimulation at 24 and 72 hours of *in vivo *ischemia.** Representative gelatin zymogram showing MMP-9 release in supernatant **(a)** and bar graphs representing mean values and SEM of 92 kDa proMMP-9 content (arbitrary unit by densitometry) **(b)**. n = 4 per group, *P <0.05 versus PBS in control conditions; #*P* <0.05 versus G-CSF in ischemia/reperfusion conditions. CON: control group; GCSF: in vivo GCSF-administered group; IS, in vivo ischemia-reperfusion group; IS GCSF: in vivo ischemia-reperfusion group with GCSF administration; MM, molecular marker, PBS: non tPA stimulated control group, SEM standard error of the mean.

**Figure 5:**
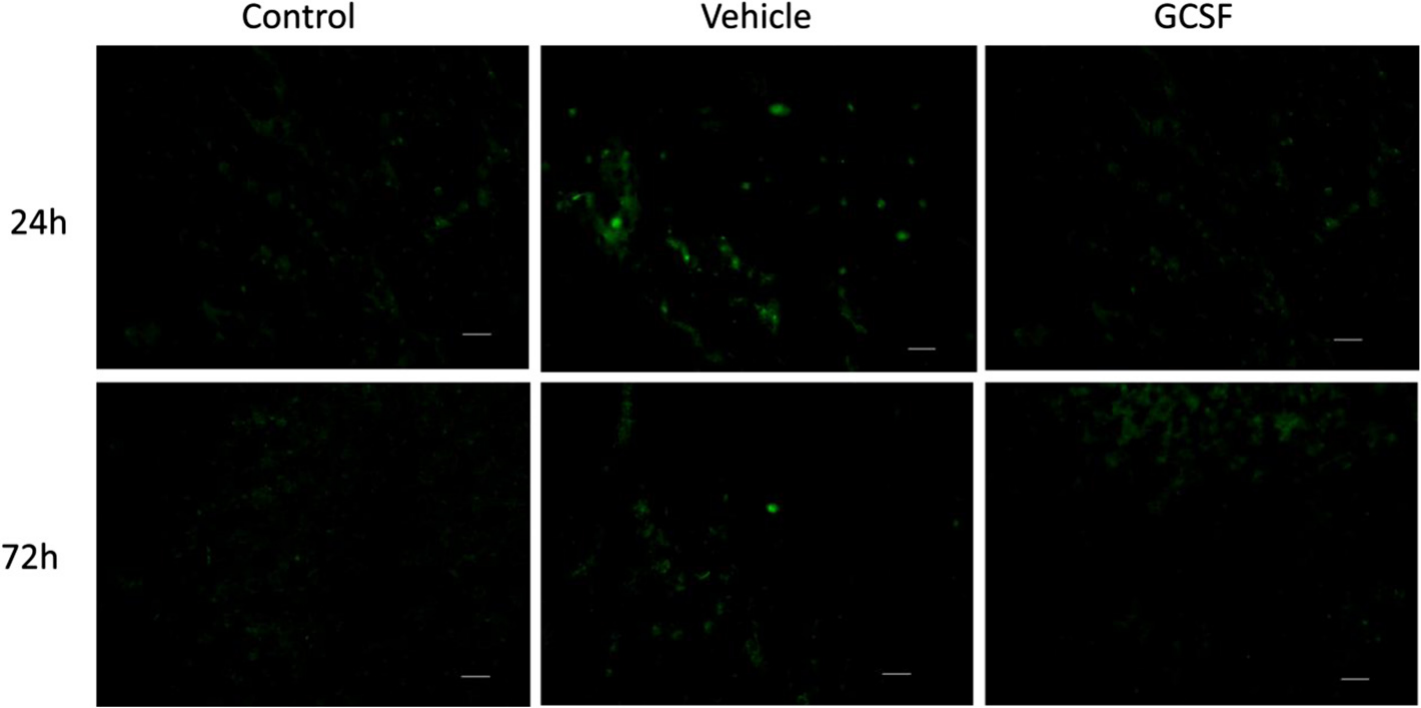
**Representative immunochemistry images of MMP-9 positive signals in the cortical infarct region in rats submitted to ischemia/reperfusion, tPA treatment and 24 or 72 hours of reperfusion.** Rats were administered with G-CSF (60 μg/kg; 30 μL) or vehicle (NaCl 0.9%, 30 μL) five hours after reperfusion (n = 5 in each group). Scale bar: 50 μm. G-CSF, granulocyte colony stimulating factor; MMP-9, matrix metallopeptidase 9; tPA, tissue plasminogen activator.

MMP-9 intensity in the supernatant of *ex vivo* tPA-treated peripheral neutrophils significantly varied according to the *in vivo* conditions (Figure 4a). Twenty four hours after the surgery, e*x vivo* tPA significantly induced the release of MMP-9 from neutrophils stemming from sham-operated rats, without a specific effect from G-CSF (*P* <0.05; Figure 4b). After ischemia/reperfusion, e*x vivo* tPA induced a significant increase in MMP-9 intensity in the supernatant of PMNs submitted *in vivo* to ischemia and G-CSF treatment (*P* <0.05). At 72 hours, no increased in MMP-9 intensity was observed in the supernatant of PMNs submitted *in vivo* to ischemia/reperfusion or ischemia/reperfusion and G-CSF treatment.In the brains of control animals there was no expression of MMP-9 at 24 and 72 hours. The MMP-9 positive signal was markedly enhanced in the infarct region after MCAO and tPA at 24 hours, and to a lesser extent at 72 hours (Figure 5). When G-CSF was administered the MMP-9 positive signal in the infarct region was no longer present at 24 and 72 hours.

## Discussion

We demonstrated for the first time that G-CSF administered in combination with tPA during the acute phase of cerebral ischemia worsened the risk of hemorrhage. Moreover, G-CSF has a harmful vascular effect witnessed by the altered endothelium-dependent relaxation (an indirect measure of more distal arteriolar damage) in the absence of the ischaemic background. This effect may be related to G-CSF's action on peripheral polynuclear neutrophils, as suggested by the absence of endothelial damage after neutrophil depletion. The increased hemorrhagic risk observed when G-CSF is associated with tPA during the acute phase of ischemia could be directly related to MMP-9 degranulation from these peripheral neutrophils. Our present work confirmed the acute neuroprotective effect of G-CSF when administered during cerebral ischemia in MCAO models [2,14,15].

This neuroprotective effect is probably independent from G-CSF’s action on neutrophils and directly related to G-CSF’s effect on neurons: previous reported mechanisms include direct neuronal impact though specific receptors, inducing neurogenesis, anti-inflammatory, and anti-apoptotic mechanisms [15-17]. The systemic anti-inflammatory effect probably explains the decreased parenchymatous infiltration of polynuclear neutrophils and the reduced presence of MMP-9 in the infarct region in spite of G-CSF-increased circulating peripheral neutrophils. In fact, in our study and according to others studies, the infiltration of neutrophils is significantly reduced after G-CSF administration; leukocytosis and MMP-9 release are restricted to the vessel compartment and do not contribute to an exacerbation of brain lesion [18,19]. As the accumulation of neutrophils within ischemic brain territories correlates with the severity of neurological injury [20], this decrease in infiltrating neutrophils could in part support the neuroprotective effect of G-CSF.

In contrast, increased neutrophils in peripheral blood due to G-CSF probably have a deleterious vascular impact. In fact, extravasation of red blood cells (the starting point of hemorrhages) correlates with changes in arteriolar endothelial cells, underpinned by degradation of the basal membrane and rupture of the blood-brain barrier [21]. We have previously demonstrated that induced-neutropenia is associated with a partial prevention of post-tPA hemorrhages, explained by the preservation of vascular endothelial function and a decrease in circulating polynuclear neutrophils [6]. It is well established that the rolling and adhesion of circulating polynuclear neutrophils during ischemia are responsible for vascular alterations in the ischaemic area [22]. Whalen *et al*. correlated the increased neutrophil count induced by G-CSF treatment to blood-brain barrier damage [23]. These circulating neutrophils are a source of MMP-9, a protease which is particularly present in and around cerebral microvessels after a brain infarct [24]. This protease could mediate blood-brain barrier breakdown, tissue injury, edema formation, and inflammation [22]. The activation of this protease is the main mechanism correlated with the risk of cerebral hemorrhage [25,26]. Therefore, G-CSF-induced leukocytosis could be deleterious for vascular endothelium and could explain the increase risk of hemorrhages.

Moreover, the MMP-9 release from PMNs could be directly stimulated and up-regulated by tPA [12,27]. This effect, documented in our study with *ex vivo* tPA treatment on cells submitted to *in vivo* ischemia, was observed at 24 hours after ischemia and no longer observed at 72 hours, in correlation with the kinetic of neutrophil proliferation, recruitment, and activation during stroke [28,29]. In our study, degranulation of MMP-9 from peripheral neutrophils was significantly increased when G-CSF was administered in the acute phase of ischemia, suggesting the combination of a direct action of G-CSF on neutrophil proliferation and activation with a direct effect of tPA on the neutrophil degranulation of MMP-9. Also worthy of note, ischemia by itself leads to rapid MMP-9 release from neutrophil degranulation [30], explaining the low level of MMP-9 release after *ex vivo* tPA at 24 hours post-ischemia in comparison to the level observed in control group. These three conditions (ischemia, tPA administration, and G-CSF-induced leukocytosis) could directly concur with the hemorrhagic risk associated with thrombolysis.

## Conclusion

Our work highlights the potential hemorrhagic risk in administering G-CSF in combination with tPA during the acute phase of cerebral ischemia, probably though vascular alterations mediated by the MMP-9 release from peripheral neutrophils.

## Abbreviations

Ach: acetylcholine; BSA: bovine serum albumin; G-CSF: granulocyte colony-stimulating factor; HT: hemorrhagic transformation; MCA: middle cerebral artery; MCAO: middle cerebral artery occlusion; MMP-9: matrix metalloproteinase; MPO: myeloperoxidase; PBS: phosphate buffer saline; PMN: polymorphonuclear neutrophils; SEM: standard error of the mean; SHR: spontaneously hypertensive rat; tPA: tissue plasminogen activator.

## Competing interests

The authors declare that they have no competing interests.

## Authors’ contributions

SG participated to the conception of the study, carried out the surgical procedure, the immunohistological evaluation, and drafted the manuscript. TO participated in the conception of the study, carried out the vascular reactivity, the immunohistological evaluation, and drafted the manuscript. MT and AD carried out the MMP-9 immunoassays. CL and ML participated in the surgical procedures and the histological evaluation. OP participated in the study of vascular reactivity and performed the statistical analysis. DL participated in the design of the study. RB conceived of the study, participated in its design and coordination, and helped to draft the manuscript. All authors read and approved the final manuscript.

## References

[B1] Minnerup J Heidrich J Wellmann J Rogalewski A Schneider A Schäbitz WR Meta-analysis of the efficacy of granulocyte-colony stimulating factor in animal models of focal cerebral ischemia Stroke 2008 39 1855 1861 10.1161/STROKEAHA.107.506816 18403735

[B2] Bråtane BT Bouley J Schneider A Bastan B Henninger N Fisher M Granulocyte-colony stimulating factor delays PWI/DWI mismatch evolution and reduces final infarct volume in permanent-suture and embolic focal cerebral ischemia models in the rat Stroke 2009 40 3102 3106 10.1161/STROKEAHA.109.553958 19644069

[B3] Sevimli S Diederich K Strecker JK Schilling M Klocke R Nikol S Kirsch F Schneider A Schäbitz WR Endogenous brain protection by granulocyte-colony stimulating factor after ischemic stroke Exp Neurol 2009 217 328 335 10.1016/j.expneurol.2009.03.018 19332060

[B4] Bath PM Sprigg N England T Colony stimulating factors (including erythropoietin, granulocyte colony stimulating factor and analogues) for stroke Cochrane Database Syst Rev 2013 6 CD005207 23797623 10.1002/14651858.CD005207.pub4 PMC11441151

[B5] Hacke W Kaste M Bluhmki E Brozman M Dávalos A Guidetti D Larrue V Lees KR Medeghri Z Machnig T Schneider D von Kummer R Wahlgren N Toni D for the ECASS Investigators Thrombolysis with alteplase 3 to 4.5 hours after acute ischemic stroke N Engl J Med 2008 359 1317 1329 10.1056/NEJMoa0804656 18815396

[B6] Gautier S Ouk T Petrault O Caron J Bordet R Neutrophils contribute to intracerebral hemorrhages after treatment with recombinant tissue plasminogen activator following cerebral ischaemia Br J Pharmacol 2009 156 673 679 10.1111/j.1476-5381.2009.00068.x 19210512 PMC2697703

[B7] Gautier S Petrault O Gele P Laprais M Bastide M Bauters A Deplanque D Jude B Caron J Bordet R Involvement of thrombolysis in recombinant tissue plasminogen activator-induced cerebral hemorrhages and effect on infarct volume and postischemic endothelial function Stroke 2003 34 2975 2979 10.1161/01.STR.0000101914.62066.7B 14615621

[B8] Niessen F Hilger T Hoehn M Hossmann KA Differences in clot preparation determine outcome of recombinant tissue plasminogen activator treatment in experimental thromboembolic stroke Stroke 2003 34 2019 2024 10.1161/01.STR.0000080941.73934.30 12843350

[B9] Matsuo Y Onodera H Shiga Y Nakamura M Ninomiya M Kihara T Tamatani T Miyasaka M Kogure K Correlation between myeloperoxidase-quantified neutrophil accumulation and ischemic brain injury in the rat Effects Neutrophil Depletion Stroke 1994 25 1469 1475 10.1161/01.str.25.7.1469 8023364

[B10] Pétrault O Ouk T Gautier S Laprais M Gelé P Bastide M Bordet R Pharmacological neutropenia prevents endothelial dysfunction but not smooth muscle functions impairment induced by middle cerebral artery occlusion Br J Pharmacol 2005 144 1051 1058 10.1038/sj.bjp.0706124 15700030 PMC1576087

[B11] Dupuis F Atkinson J Limiñana P Chillon JM Captopril improves cerebrovascular structure and function in old hypertensive rats Br J Pharmacol 2005 144 349 356 10.1038/sj.bjp.0706001 15655534 PMC1576005

[B12] Cuadrado E Ortega L Hernández-Guillamon M Penalba A Fernández-Cadenas I Rosell A Montaner J Tissue plasminogen activator (t-PA) promotes neutrophil degranulation and MMP-9 release J Leukoc Biol 2008 84 207 214 10.1189/jlb.0907606 18390930

[B13] Roos D De Boer M Purification and cryopreservation of phagocytes from human blood Methods Enzymol 1986 132 225 243 3821511 10.1016/s0076-6879(86)32010-x

[B14] Six I Gasan G Mura E Bordet R Beneficial effect of pharmacological mobilization of bone marrow in experimental cerebral ischemia Eur J Pharmacol 2003 458 327 328 10.1016/S0014-2999(02)02785-1 12504790

[B15] Schneider A Krüger C Steigleder T Weber D Pitzer C Laage R Aronowski J Maurer MH Gassler N Mier W Hasselblatt M Kollmar R Schwab S Sommer C Bach A Kuhn HG Schäbitz WR The hematopoietic factor G-CSF is a neuronal ligand that counteracts programmed cell death and drives neurogenesis J Clin Invest 2005 115 2083 2098 10.1172/JCI23559 16007267 PMC1172228

[B16] Minnerup J Sevimli S Schäbitz WR Granulocyte-colony stimulating factor for stroke treatment: mechanisms of action and efficacy in preclinical studies Exp Transl Stroke Med 2009 1 2 10.1186/2040-7378-1-2 20142989 PMC2816868

[B17] Solaroglu I Cahill J Tsubokawa T Beskonakli E Zhang JH Granulocyte colony-stimulating factor protects the brain against experimental stroke via inhibition of apoptosis and inflammation Neurol Res 2009 31 167 172 10.1179/174313209X393582 19298757

[B18] Sehara Y Hayashi T Deguchi K Zhang H Tsuchiya A Yamashita T Lukic V Nagai M Kamiya T Abe K Decreased focal inflammatory response by G-CSF may improve stroke outcome after transient middle cerebral artery occlusion in rats J Neurosci Res 2007 85 2167 2174 10.1002/jnr.21341 17497673

[B19] Strecker JK Sevimli S Schilling M Klocke R Nikol S Schneider A Schäbitz WR Effects of G-CSF treatment on neutrophil mobilization and neurological outcome after transient focal ischemia Exp Neurol 2010 222 108 113 10.1016/j.expneurol.2009.12.012 20026112

[B20] Buck BH Liebeskind DS Saver JL Bang OY Yun SW Starkman S Ali LK Kim D Villablanca JP Salamon N Razinia T Ovbiagele B Early neutrophilia is associated with volume of ischemic tissue in acute stroke Stroke 2008 39 355 360 10.1161/STROKEAHA.107.490128 18162626

[B21] Dijkhuizen RM Asahi M Wu O Rosen BR Lo EH Rapid breakdown of microvascular barriers and subsequent hemorrhagic transformation after delayed recombinant tissue plasminogen activator treatment in a rat embolic stroke model Stroke 2002 33 2100 2104 10.1161/01.STR.0000023534.37670.F7 12154270

[B22] Del Zoppo GJ Mabuchi T Cerebral microvessel responses to focal ischemia J Cereb Blood Flow Metab 2003 23 879 894 12902832 10.1097/01.WCB.0000078322.96027.78

[B23] Whalen MJ Carlos TM Wisniewski SR Clark RS Mellick JA Marion DW Kochanek PM Effect of neutropenia and granulocyte colony stimulating factor-induced neutrophilia on blood–brain barrier permeability and brain edema after traumatic brain injury in rats Crit Care Med 2000 28 3710 3717 10.1097/00003246-200011000-00029 11098978

[B24] Rosell A Cuadrado E Ortega-Aznar A Hernández-Guillamon M Lo EH Montaner J MMP-9-positive neutrophil infiltration is associated to blood–brain barrier breakdown and basal lamina type IV collagen degradation during hemorrhagic transformation after human ischemic stroke Stroke 2008 39 1121 1126 10.1161/STROKEAHA.107.500868 18323498

[B25] Castellanos M Leira R Serena J Pumar JM Lizasoain I Castillo J Davalos A Plasma metalloproteinase-9 concentration predicts hemorrhagic transformation in acute ischemic stroke Stroke 2003 34 40 46 10.1161/01.STR.0000046764.57344.31 12511748

[B26] Montaner J Molina CA Monasterio J Abilleira S Arenillas JF Ribó M Quintana M Alvarez-Sabín J Matrix metalloproteinase-9 pretreatment level predicts intracranial hemorrhagic complications after thrombolysis in human stroke Circulation 2003 107 598 603 10.1161/01.CIR.0000046451.38849.90 12566373

[B27] Ning M Furie KL Koroshetz WJ Lee H Barron M Lederer M Wang X Zhu M Sorensen AG Lo EH Kelly PJ Association between tPA therapy and raised early matrix metalloproteinase-9 in acute stroke Neurology 2006 66 1550 1555 10.1212/01.wnl.0000216133.98416.b4 16717217

[B28] Jin R Yang G Li G Inflammatory mechanisms in ischemic stroke: role of inflammatory cells J Leukoc Biol 2010 87 779 789 10.1189/jlb.1109766 20130219 PMC2858674

[B29] Grønberg NV Johansen FF Kristiansen U Hasseldam H Leukocyte infiltration in experimental stroke J Neuroinflammation 2013 10 115 10.1186/1742-2094-10-115 24047275 PMC3852747

[B30] Justicia C Panés J Solé S Cervera A Deulofeu R Chamorro A Planas AM Neutrophil infiltration increases matrix metalloproteinase-9 in the ischemic brain after occlusion/reperfusion of the middle cerebral artery in rats J Cereb Blood Flow Metab 2003 23 1430 1440 14663338 10.1097/01.WCB.0000090680.07515.C8

